# A Mechanistic Site-Of-Action Model: A Tool for Informing Right Target, Right Compound, And Right Dose for Therapeutic Antagonistic Antibody Programs

**DOI:** 10.3389/fbinf.2021.731340

**Published:** 2021-09-03

**Authors:** Georgi I. Kapitanov, Jeffrey R. Chabot, Jatin Narula, Mahua Roy, Hendrik Neubert, Joe Palandra, Vahid Farrokhi, Jay S. Johnson, Robert Webster, Hannah M. Jones

**Affiliations:** ^1^ BioMedicine Design, Pfizer Worldwide R&D, Cambridge, MA, United States; ^2^ BioMedicine Design, Pfizer Worldwide R&D, Andover, MA, United States

**Keywords:** site of action, PKPD, QSP, modeling and simulation, LC-MS

## Abstract

Quantitative modeling is increasingly utilized in the drug discovery and development process, from the initial stages of target selection, through clinical studies. The modeling can provide guidance on three major questions–is this the right target, what are the right compound properties, and what is the right dose for moving the best possible candidate forward. In this manuscript, we present a site-of-action modeling framework which we apply to monoclonal antibodies against soluble targets. We give a comprehensive overview of how we construct the model and how we parametrize it and include several examples of how to apply this framework for answering the questions postulated above. The utilities and limitations of this approach are discussed.

## Introduction

Modeling and simulation tools have become an essential part of the drug development process (Norris et al., 2000; Lalonde et al., 2007; Barrett et al., 2008; Edginton et al., 2008; Mager and Jusko, 2008; Rajman, 2008; Jones et al., 2009; Allerheiligen, 2010; van der Graaf and Benson, 2011; Zhao et al., 2011; Jones et al., 2012a; Knight-Schrijver et al., 2016; Danhof et al., 2018; Nijsen et al., 2018). Applying quantitative analyses early in the drug discovery can be very informative for selecting and de-selecting those programs with the best/least chance of clinical success. Traditional pharmacokinetics/pharmacodynamics (PKPD) models, while still widely utilized (Meibohm and Derendorf, 1997; Derendorf and Meibohm, 1999; Rajman, 2008), have gradually given place to increased mechanistic modeling complexity, with the intent to achieve higher predictive accuracy and mechanistic insights. These mechanistic modeling techniques include systems biology (SB) (Kitano, 2005; Kohl et al., 2010; Benson et al., 2011), quantitative systems pharmacology (QSP) (Hopkins, 2008; Allerheiligen, 2010; van der Graaf and Benson, 2011; van der Graaf, 2012; Jusko, 2013; Rogers et al., 2013; Peterson and Riggs, 2015; Knight-Schrijver et al., 2016; Danhof et al., 2018; Nijsen et al., 2018; Cucurull-Sanchez et al., 2019), and physiologically based pharmacokinetics (PBPK) (Baxter et al., 1994; Andersen, 1995; Baxter et al., 1995; Hoang, 1995; Arundel, 1997; Blakey et al., 1997; Nestorov et al., 1998; Grass and Sinko, 2002; Aarons, 2005; Jones et al., 2006a; Jones et al., 2006b; Cai et al., 2006; Barton et al., 2007; Nestorov, 2007; Edginton et al., 2008; Loizou et al., 2008; Jones et al., 2009; Chabot et al., 2011; Jones et al., 2011; Jones et al., 2012a; Jones et al., 2012b; Bouzom et al., 2012; Huang and Rowland, 2012; Rostami-Hodjegan et al., 2012; Shah and Betts, 2012; Zhao et al., 2012; Jones et al., 2013). Traditional empirical PKPD models are useful in predicting dosing and estimating pharmacology/efficacy in later stage clinical development and translation from pre-clinical animal models to humans. However, an extensive amount of pre-clinical PKPD data is needed to utilize them which limits their ability to be used to make early pre-clinical recommendations, before lead candidates are defined. In contrast, the mechanistic models, while often complex and computationally intensive, appear more suitable for overall disease and molecule modality recommendations.

Early stage biologics drug discovery programs concern themselves with three main questions: (1) is the proposed target biologically relevant and is hitting the target feasible; (2) what are the drug characteristics that would allow for biomarker modulation or efficacy; (3) what is the efficacious dose in humans? These three questions can be summarized as three components to each project: right target, right compound, right dose. Considering the high number of potential new targets, a flexible mechanistic modeling framework is needed that can be used to perform sensitivity analysis on a discrete number of parameters. This approach would quickly pinpoint gaps in knowledge that can be tested experimentally and make timely recommendations for each of the three components of project development. Therefore, for this purpose, one needs a model that is on the spectrum of complexity somewhere between the traditional PKPD and the multiscale systems biology models. We propose a site-of-action (SoA) model for assisting in the discovery and development of biologics.

The site-of-action model extends a two compartment PKPD model by including the mechanistic interactions of the drug and its target (e.g., binding, unbinding and drug-target complex clearance), the relevant properties of the target, as well as a separate compartment that models the tissues in which the disease progresses (a so-called site of action) (Brodfuehrer et al., 2014). Such a model can be implemented rapidly since it captures only the relevant biology and is expressed through a discrete number of differential equations, variables, and parameters, which allows for extensive sensitivity analysis to identify the important parameters and biological assumptions that need to be investigated further. Hence, this model should be considered a starting point from which to build out specific models of the biology of different targets, its main utility being in early stage projects.

A previous iteration of the model has been described by Tiwari et al. 2016a and used for assessing sensitivity of the projected target neutralization to target concentrations (Tiwari et al., 2016a) and antibody affinity (Tiwari, et al, 2017). The current iteration makes minor changes to the old model structure and goes into more detail in explaining the reasons for certain modeling and parameter value choices. We have implemented this approach successfully since, and, beyond the theoretical treatment in Tiwari et al. 2016a, in Applications of SoA Model Methodology of this manuscript will be demonstrating its utility by several real-world examples. The modeling work is highly dependent on robust assays to inform the parametrization of the model (biomeasures), which is yet another important expansion to the work presented in Tiwari et al. 2016a. We have listed the typical assays and input data used in the Target Parameters section. For the purposes of this article, we will focus on soluble targets. Membrane targets deserve to be covered in a separate manuscript, both in terms of the modeling approach, as well as in terms of utilizing the range of biomeasure assays and tools for supporting the modeling efforts.

## Model Structure and Methods of Parametrization

The model is an extension of a drug-target mechanistic binding two-compartment model that accounts for the relevant disease tissue, which is referred to as site of action (SoA). Free plasma drug (with concentration $D_{P}$ in plasma volume $V_{P\;}$ ) distributes into non-specific (peripheral with volume $V_{T})$ and SoA (disease-specific with volume $V_{S})$ compartments. In plasma and at the SoA, the drug binds reversibly to target protein (with concentrations $T_{P}$ and $T_{S}$ , respectively) to form a drug-target complex (with concentrations $C_{P}$ and $C_{S}$ , respectively). The binding kinetics are characterized by a second-order association $\left(k_{on}\right)$ and first-order dissociation $\left(k_{off}\right)$ rate constants. The model assumes target synthesis and degradation both in the central and the SoA compartments (expressed by the zero order rates $ksyn_{S}$ , and $ksyn_{P}$ and first order rates $k_{degTp}$ and $k_{degTs}$ , respectively), target distribution between plasma and the SoA ( $k_{psT}$ and $k_{spT}$ ), and drug - target complex distribution between plasma and SoA ( $k_{psC}$ and $k_{spC}$ ) and elimination in plasma only ( $k_{elC}$ ). The modeling equations are:

$$\begin{array}{c} \begin{array}{c} \frac{dD_{P}}{dt}=A+\;k_{sp}D_{S}\frac{V_{S}}{V_{P}}+k_{tp}D_{T}\frac{V_{T}}{V_{P}}-k_{ps}D_{P}-k_{pt}D_{P}+k_{off}C_{P}-k_{on}D_{P}T_{P}-k_{el}D_{P} \end{array} \end{array}$$ (1)

$$\begin{array}{c} \frac{dD_{S}}{dt}=k_{ps}D_{P}\frac{V_{P}}{V_{S}}-k_{sp}D_{S}+k_{off}C_{S}-k_{on}D_{S}T_{S\;} \end{array}$$ (2)

$$\begin{array}{c} \frac{dD_{T}}{dt}=k_{pt}D_{P}\frac{V_{P}}{V_{T}}-k_{tp}D_{T} \end{array}$$ (3)

$$\begin{array}{c} \frac{dC_{P}}{dt}=k_{spC}C_{S}\frac{V_{S}}{V_{P}}-k_{psC}C_{P}+k_{on}D_{P}T_{P}-k_{off}C_{P}-k_{elC}C_{P}\; \end{array}$$ (4)

$$\begin{array}{c} \frac{dC_{S}}{dt}=k_{psC}C_{P}\frac{V_{P}}{V_{S}}-k_{spC}C_{S}+k_{on}D_{S}T_{S}-k_{off}C_{S}\; \end{array}$$ (5)

$$\begin{array}{c} \frac{dT_{P}}{dt}=k_{spT}T_{S}\frac{V_{S}}{V_{P}}-k_{psT}T_{P}+k_{off}C_{P}-k_{on}D_{P}T_{P}+\;k_{synP}-k_{degTp}T_{P}\; \end{array}$$ (6)

$$\begin{array}{c} \frac{dT_{S}}{dt}=k_{psT}T_{P}\frac{V_{P}}{V_{S}}-k_{spT}T_{S}+k_{off}C_{S}-k_{on}D_{S}T_{S}+k_{synS}-k_{degTs}T_{P} \end{array}$$ (7)

where $D_{i}$ , $C_{i}$ and $T_{i}$ represent the concentrations of free drug, drug-target complex, and free target in plasma (i=P) , or SoA (i=S) compartment, respectively. $D_{T}$ is the free drug concentration in the peripheral tissue compartment. A is the drug influx function, which is administration dependent.

**FIGURE 1:**
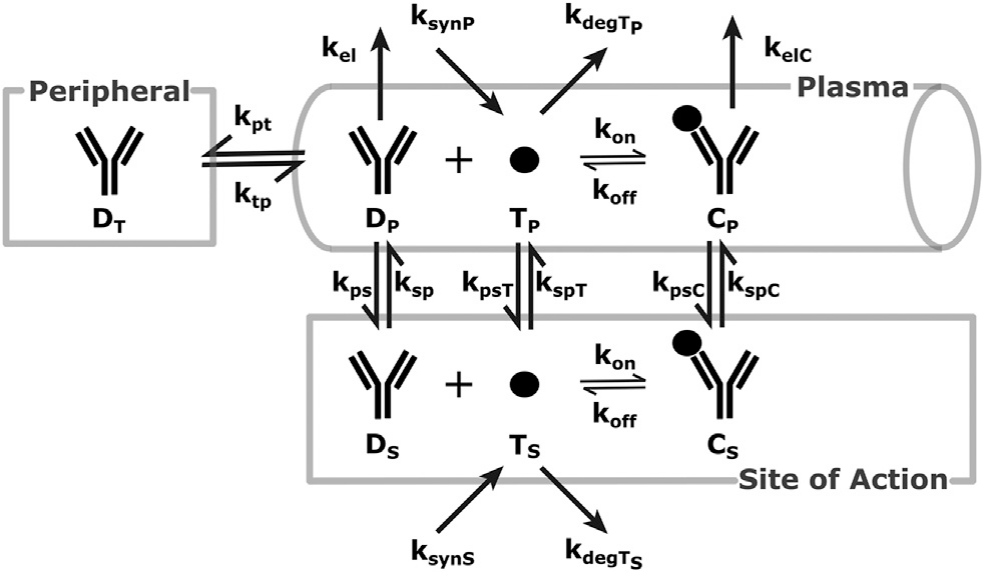
SoA Model Scheme: A diagram describing the distribution and elimination of the mAb (denoted by D), synthesis, distribution and elimination of the target (denoted by T) and the interactions between the mAb and target and the distribution and elimination of the resulting mAb:target complex (denoted by C). Subscripts describe the compartments - p for plasma, t for peripheral tissues, s for the site of action. Detailed descriptions of all variables and parameters are in Model Structure and Methods of Parametrization and Table 1.

A schematic of the modeling reactions is shown in Figure 1. Table 1 lists the variables and parameters contained in the system of differential equations, with explanations.

**TABLE 1:** Definition of parameters used in SoA model.

Parameter	Description	Value	Units
$V_{P}$	Central compartment volume (plasma)	Drug specific	L
Q	Drug distributive clearance rate	Drug specific	L/day
Cl	Drug elimination clearance rate	Drug specific	L/day
$V_{S}$	Volume of SoA interstitial space	Tissue specific	L
$V_{T}$	Peripheral tissue volume (calculated)	Model specific	L
$V_{2}$	Peripheral tissue volume (from two-compartment PK)	Drug specific	L
$D_{0}$	Dose	Study specific	Nanomole
$k_{a}$	Rate of absorption post subcutaneous drug administration	Drug specific	1/day
F	Bioavailability post subcutaneous drug administration	Drug specific	Dimensionless
$Q_{target}$	Target distributive clearance rate	Target specific	L/day
ratio	Ratio of plasma versus SoA drug concentrations at steady state	SoA specific	Dimensionless
$k_{on}$	Drug-target concentration-dependent association rate	Drug specific	nM^−1^day^−1^
$k_{ps}$	Rate constant of drug distribution from plasma to SoA	$ratio*\frac{Q}{V_{2}}*\frac{V_{S}}{V_{P}}$	1/day
$k_{sp}$	Rate constant of drug distribution from SoA to plasma	$\frac{Q}{V_{2}}$	1/day
$k_{pt}$	Rate constant of drug distribution from plasma to peripheral tissue	$\frac{Q}{V_{2}}-k_{ps}$	1/day
$k_{tp}$	Rate constant of drug distribution from peripheral tissue to plasma	$\frac{Q}{V_{2}}$	1/day
$k_{psT}$	Rate constant of target distribution from plasma to SoA	$\frac{Q_{target}}{V_{P}}$	1/day
$k_{spT}$	Rate constant of target distribution from SoA to plasma	Calculated to ensure target equilibrium in absence of drug	1/day
$k_{psC}$	Rate constant of complex distribution from plasma to SoA	$k_{ps}$	1/day
$k_{spC}$	Rate constant of complex distribution from SoA to plasma	$k_{sp}$	1/day
$k_{el}$	Rate constant of drug elimination from plasma	$\frac{Cl}{V_{P}}$	1/day
$k_{degTp}$	Rate constant of target elimination from plasma	Target specific	1/day
$k_{degTs}$	Rate constant of target elimination from SoA	Target specific	1/day
$k_{elC}$	Rate constant of complex elimination from plasma	For soluble target can be assumed = $k_{el}$ , unless data are avalable	1/day
$k_{off}$	First-order dissociation rate constant of antibody	$k_{on}*K_{D}$	1/day
$k_{synP}$	Zero order target synthesis rate in plasma	Calculated to ensure target equilibrium in absence of drug	nM/day
$k_{synS}$	Zero order target synthesis rate in SoA	Calculated to ensure target equilibrium in absence of drug	nM/day

The initial conditions of the variables above appear with a 0 after the subscript: $D_{P0}$ is initial drug concentration in the plasma, $T_{S0}$ is initial target concentration at the SoA, etc.

Table 2 presents the value of A and initial conditions related to the drug variables dependent on administration route. Other administration routes can be incorporated as well.

**TABLE 2:** Expressions for A depending on route of drug administration.

Route of administration	Expression for A	Drug-related initial conditions
Intravenous bolus	A=0	$D_{P0}=D_{0},\;D_{T0}=\;D_{S0}=0$
Subcutaneous	$A=\;D_{0}*F*k_{a}$	$D_{P0}=\;D_{T0}=\;D_{S0}=0$

## Parameter Determination

The next few sections will discuss how the different parameters are estimated and suggest typical assumptions for their values. Initial parametrization of the model may be obtained from the literature or data repositories. However, when such information is unavailable, experimental determination of key model parameters may be required. Some of the experimental methods discussed here may not be applicable, appropriate, or even possible for particular targets. A conversation among modelers, biologists, and biomeasure analysts would determine the most appropriate path to appropriate parametrization of the model.

### Drug Distribution Parameters

Monoclonal antibody (mAb) PK typically shows biphasic behavior and such data can be modeled using two-compartmental models, resulting in the estimation of 4 PK parameters (e.g., V1 , Cl , Q  and V2 ) (Betts et al., 2018). A question then arises–what is the concentration of antibody at the SoA? There have been several preclinical studies that have been performed to measure concentrations in tissues relative to blood across different antibodies (Vugmeyster et al., 2008; Vugmeyster et al., 2010; Shah and Betts, 2013; Li et al., 2017; An et al., 2020). Antibodies distribute predominantly in the interstitium of tissues (Janeway and Walport, 2001), therefore the concentration ratio needs to be adjusted for the interstitial volume of the tissue of interest. Generally, the volume of the interstitium is around 1/3 of the total tissue volume (Poulin and Theil, 2002) (which includes peripheral blood and cells), unless one deals with certain specific organs like muscles or the brain (Shah and Betts, 2013). The ratio of total tissue to serum concentration for most organs in preclinical species is around 10% (Shah and Betts, 2013; Vugmeyster et al., 2010). Therefore, our recommendation is to use 30% (=10%/1/3) as a standard ratio parameter for non-brain and non-muscle tissue SoAs.

The following method can account for any ratio deemed appropriate for the particular project.

The calculations for the drug distribution constants presented here have two simultaneous aims: to retain mAb plasma PK and maintain the average concentration ratio (expressed as the parameter ratio ) between the SoA and the plasma compartments (ratio of areas under the curve (AUCs) is equivalent). The following relationships are derived based on steady state analysis of total mAb concentration pharmacokinetics:

$$\begin{array}{c} {\begin{array}{c} k_{ps}=ratio\;k_{sp}\frac{V_{S}}{V_{P}} \end{array}}_{\;}\; \end{array}$$ (8)

$$\begin{array}{c} k_{pt}=\;\frac{\;Q}{V_{P}}-k_{ps}\; \end{array}$$ (9)

$$\begin{array}{c} V_{T}=\;\frac{V_{P}\;k_{pt}\;}{k_{tp}}, \end{array}$$ (10)

where

$$\;k_{sp}=\;k_{tp}=\;\frac{\;Q}{V_{2}}\;.$$

There is an important distinction between $V_{2}$ and $V_{T}$ which warrants further elaboration. Fixing the ratio of average drug concentration at the SoA vs the plasma, while preserving plasma PK, necessitates an extra degree of freedom in the calculations. Since drug concentrations in the peripheral tissue are rarely of interest, the peripheral tissue volume is a convenient (and mathematically sound) choice. However, the calculations of the drug distribution rates are done with the peripheral tissue volume of distribution from the PK parameter estimates, $V_{2}$ (see Table 1). In our practice, $V_{T}$ is not used and peripheral mAb concentrations not tracked (as opposed to SoA concentrations), but understanding of the mathematics behind the model would be incomplete without $V_{T}$ ’s explicit inclusion in the equations and the parameter set. Still, the reader’s ability to use the model would not be inhibited by ignoring this extra mathematics.

### Binding Parameters

If $k_{on}$ has not been determined by a surface plasmon resonance method (Tang et al., 2010), or other methods, such as KinExA (Wani et al., 2016), $k_{on}$ can be assumed to be $1e6\;M^{-1}s^{-1}$ (Foote and Eisen, 1995). Given a measured constant of dissociation ( $K_{D}$ ), one can calculate $k_{off}$ as the product of $k_{on}$ and $K_{D}$ . Drug:target interactions in the peripheral compartment and complex distribution from plasma to the peripheral compartment are typically ignored. To make binding interactions even more mechanistic, one can include step-wise binding for each of the antibody’s arm. In this case the binding interactions from Eqs 1–7 would need to be rewritten as follows:

$$\begin{array}{c} \frac{dD_{P}}{dt}=\ldots +k_{off}C_{P}-2\;k_{on}D_{P}T_{P}\; \end{array}$$ (11)

$$\begin{array}{c} \frac{dD_{S}}{dt}=\ldots +k_{off}C_{S}-2\;k_{on}D_{S}T_{S}\; \end{array}$$ (12)

$$\begin{array}{c} \frac{dC_{P}}{dt}=\ldots +2\;k_{on}D_{P}T_{P}-k_{off}C_{P}-k_{on}C_{P}T_{P}+2\;k_{off}C_{P2} \end{array}$$ (13)

$$\begin{array}{c} \frac{dC_{S}}{dt}=\ldots +2\;k_{on}D_{S}T_{S}-k_{off}C_{S}-k_{on}C_{S}T_{S}+2\;k_{off}C_{S2} \end{array}$$ (14)

$$\begin{array}{c} \frac{dT_{P}}{dt}=\ldots +k_{off}C_{P}-2\;k_{on}D_{P}T_{P}-k_{on}C_{P}T_{P}+2\;k_{off}C_{P2} \end{array}$$ (15)

$$\begin{array}{c} \frac{dT_{S}}{dt}=\ldots +k_{off}C_{S}-2\;k_{on}D_{S}T_{S}-k_{on}C_{S}T_{S}+2\;k_{off}C_{S2} \end{array}$$ (16)

$$\begin{array}{c} \frac{dC_{P2}}{dt}=k_{spC}C_{S2}\frac{V_{S}}{V_{P}}-k_{psC}C_{P2}+k_{on}D_{P}T_{P}-k_{off}C_{P}-k_{elC}C_{P2} \end{array}$$ (17)

$$\begin{array}{c} \frac{dC_{S2}}{dt}=k_{psC}C_{P2}\frac{V_{P}}{V_{S}}-k_{spC}C_{S2}+k_{on}D_{S}T_{S}-k_{off}C_{S2} \end{array}$$ (18)

For variables $D_{P}$ , $D_{S}$ , $T_{P}$ , $T_{S}$ , $C_{P}$ , $C_{S}$ , the only differences between Eqs 11–18 and Eqs 1–7 are the binding interactions, hence synthesis, distribution, and elimination reactions are replaced by ellipses for simplicity. Two new species are introduced: $C_{P2}$ and $C_{S2}$ , which represent the concentration of mAb bound to its target on each arm (a double complex) in plasma and the SoA, respectively. The factors of two account for the multiple ways in which an unbound antibody can engage a target, or a doubly bound antibody can release a target. Whether to include this mechanistic binding step is dependent on the biology and the requirement for this extra complexity.

### Target Parameters

The target related parameters are turnover (half-life or degradation rate), synthesis rate, concentrations (both in plasma and the SoA) and distribution rate (between the plasma and the SoA).

#### Estimating Target Concentrations

There are many published methods or approaches for measuring target levels both in serum and tissue (Becker and Hoofnagle, 2012), including ligand binding assays and mass spectrometry. A preferred approach, as previously highlighted due to its enhanced specificity and selectivity, is protein or peptide immunoaffinity liquid chromatography tandem mass spectrometry (IA LC-MS/MS). The method requires selection of optimal capture reagents, calibration standards and surrogate peptides for detection. In this method, proteins and/or trypsin digested peptides are enriched by anti-protein or anti-peptide antibodies or a sequential combination of both approaches. The enriched peptides are quantified using detection by nanoflow LC-MS/MS. A detailed description of this method can be found in Palandra et al. (2013) and Neubert et al. (2020). The addition of a stable isotope-labeled synthetic version of the surrogate peptide(s) prior to protein digestion reduces variables and quantitation relative to the chosen protein calibrator can be achieved. The mass spectrometric response of the endogenous peptide is compared to the analogous response for the labelled peptide in all samples, including calibrators, thereby normalizing for digestion efficiency and matrix suppression differences between the samples (Bantscheff et al., 2007). Examples of well designed, fit for purpose, sequential protein and tryptic peptide IA-LC-MS can achieve lower limit of quantitation (LLOQ) of sub 10 pg/ml (Neubert et al., 2013; Palandra et al., 2013), while protein IA and peptide IA only approaches are typically capable of achieving sub 100 pg/ml LLOQ (McAvoy et al., 2014; Zhao et al., 2018; Shuford et al., 2020).

Target expression levels vary widely depending on their biological function, disease state, tissue localization, and many other factors. For example, the growth and differentiation factor 8 (GDF-8), is present in circulation at very high expression levels of approximately 7 ng/ml in adult humans owing to its function in regulating muscle mass (Palandra et al., 2016). While targets like Interleukin-21 are not detected in human serum and can only be measured in certain human tissues such as colon tissue at an average concentration of 1 ng/g (Palandra et al., 2013). The alarmin cytokine, Interleukin-33 (IL-33) is present in circulation at approximately 20–100 pg/ml (Artru et al., 2020) and in many tissues at very elevated concentrations (200 ng/g in the lung (Cohen et al., 2015)) owing to its ubiquitous presence in the nucleus of all producing human cells. When the concentrations in a tissue homogenate are measured, the concentrations that are provided need to be adjusted for the interstitial volume of the analyzed tissue before being applied in the SoA model. Again, other methodologies have been used in some cases as driven by the protein and analytical complexities.

#### Estimating Target Turnover

While traditionally radio-labelling methods have been used for estimating turnover, methods based on *in-vivo* stable isotope labelling and proteomics have been established to measure physiologically relevant turnover (Bateman et al., 2006; Lindwall et al., 2006; Doherty and Whitfield, 2011; Hinkson and Elias, 2011; Lassman et al., 2014; Larance and Lamond, 2015). One of the preferred methods uses immunoaffinity enrichment of the target proteins from a stable isotope labeled amino acid tracer pulse-chase study, either from preclinical or clinical studies. Tracer incorporation in a surrogate peptide sequence is then measured by targeted mass spectrometry. The workflow and details of the study have been published (Farrokhi et al., 2018a). Once data is available for both the tracer levels and its incorporation in the protein of interest, the turnover rate is estimated using a series of models that account for the tracer’s incorporation, as well as the known biological properties of the protein of interest (e.g., a shed receptor in the tissue vs cytokine released primarily in plasma). An earlier version of these models was used in Farrokhi et al. 2018a These assays are confined by the time limitations in pulse-chase durations (multiple hours or a few days) in *in vivo* studies and accurate measurement of slow turnover rates (i.e., multiple days or weeks) are not feasible or are estimated from extrapolation. Also, in some cases, measurements are not feasible due to low concentrations of the target protein. Other methodologies have also been published in the literature, (Bateman et al., 2006; Lindwall et al., 2006; Doherty and Whitfield, 2011; Hinkson and Elias, 2011; Lassman et al., 2014; Larance and Lamond, 2015), but they are likely to experience similar limitations. Physiological target turnover measurements in human is limited to only the soluble targets and turnover in SoA is estimated from soluble target when possible.

#### Target Synthesis Rate

Generally, once information about the target’s concentrations and turnover are available, the synthesis is calculated assuming that in the absence of drug the system is at steady state. The rate constant for target distribution from the plasma to the site of action ( $\;k_{psT}$ ) can be fixed. The rate constant for target distribution from the site of action to the plasma ( $k_{spT}$ ) is derived based on the steady state levels of target in the plasma and the SoA together with $k_{psT}$ . At steady state, synthesis rates, degradation rates, and distribution rate constants between the plasma and the SoA must be balanced to achieve known levels of target concentrations in both compartments.

Target steady state concentration in plasma prior to drug administration is defined by:

$$\begin{array}{c} \frac{dT_{P}}{dt}=k_{synP}-T_{P0}\left(k_{degTp}+k_{psT}\right)+T_{S0}\;k_{spT}\;V_{S}/V_{P}=0 \end{array}$$ (19)

Target steady state concentration at the SoA prior to drug administration is defined by:

$$\begin{array}{c} \frac{dT_{S}}{dt}=k_{synS}-T_{S0}\left(k_{degTs}+k_{spT}\right)+T_{P0}\;k_{psT}\;V_{P}/V_{S}=\;0 \end{array}$$ (20)

Total target synthesis in the human body (in amount, nanomoles) is defined as:

$$\begin{array}{c} k_{synTot}=k_{synP}\;V_{p}+k_{synS}\;V_{S}=\;T_{P0}\;k_{degTp}\;V_{P}+T_{S0}\;k_{degTs}\;V_{S} \end{array}$$ (21)

It is rare that one has information about the ratio between target synthesis in plasma versus the SoA. This ratio is generally assumed taking into account what is known about the biology. For the remainder of this section and for the purpose of equations and calculations, the fraction of total synthesis in the SoA is captured by the parameter frac .

#### Estimating Target Distribution

While drug distribution constants can be calculated from the PK and Eqs 8–10 above, target rates of distribution are largely unknown. We fix $k_{psT}$ :

$$\begin{array}{c} k_{psT}=\;\frac{Q_{target}}{V_{P}}.\; \end{array}$$ (22)

At an exploratory stage, we use a parameter value for $Q_{target}$ , estimated from literature data reporting a distributive clearance rate of Albumin from Synovial joints of Rheumatoid arthritis patients (Owen et al., 1994). While this parameter value can be used in the initial stages of a project, as a project progresses, this value is explored in more detail and is updated by considering a pharmacokinetics based value for a recombinant version of the target, e.g., (Creaven et al., 1987; Banks et al., 2000; Zhang et al., 2019), or by basing $Q_{target}$ on the molecular weight of the target (Li et al., 2017).

Since these approaches have not be largely validated and adopted, one is advised to employ sensitivity analysis regarding target distribution parameters.

If $k_{psT}$ is fixed, assuming $k_{synS}=frac*\frac{k_{synTot}}{V_{S}}$ and rearranging Eqs 19 and 22 will result in:

$$\begin{array}{c} k_{spT}=frac\;\frac{\;\left(T_{P0}\;k_{degTp}\;V_{P}+T_{S0}\;k_{degTs}\;V_{S}\right)}{T_{S0}\;V_{S}}\;-\;k_{degTs}+\;\frac{T_{P0}\;V_{P}}{T_{S0}\;V_{S}}{\;}_{*\;}k_{psT} \end{array}$$ (23)

## Applications of Site-of-Action Model Methodology

This section provides four examples of application of the SoA modeling structure to soluble targets. The examples are divided into the three main categories for successful use of translational modeling and simulation: right target, right compound, and right dose.

### Right Target

In the pre-clinical space, especially in early project stages, it is appropriate to conduct feasibility analysis. At this stage, a successful assessment is both one that progresses a target as a part of the portfolio and eventually into the clinic, as well as one that shows that a target is infeasible from a clinical utility standpoint. Such analyses are performed to determine whether sufficient levels of target coverage can be achieved via neutralization with a monoclonal antibody and should not be confused with determining whether the target is “right” from a disease standpoint. Often these analyses are done with just in-vitro functional assays, whose utility is limited - they capture a narrow aspect of the biology and may be done in the pre-portfolio stage. Once the project is part of the portfolio, we recommend a more thorough analysis with a SoA model since more resources for modeling and biomeasures/biomarkers are available. In many cases, the required levels of target coverage for efficacy are unknown so a threshold is set, depending on the disease, competitive landscape, and other factors, often at >90% or >99% target neutralization. If the required coverage for pharmacology cannot be achieved at a commercially viable dose, project termination is recommended.

#### Osteopontin Example

**FIGURE 2:**
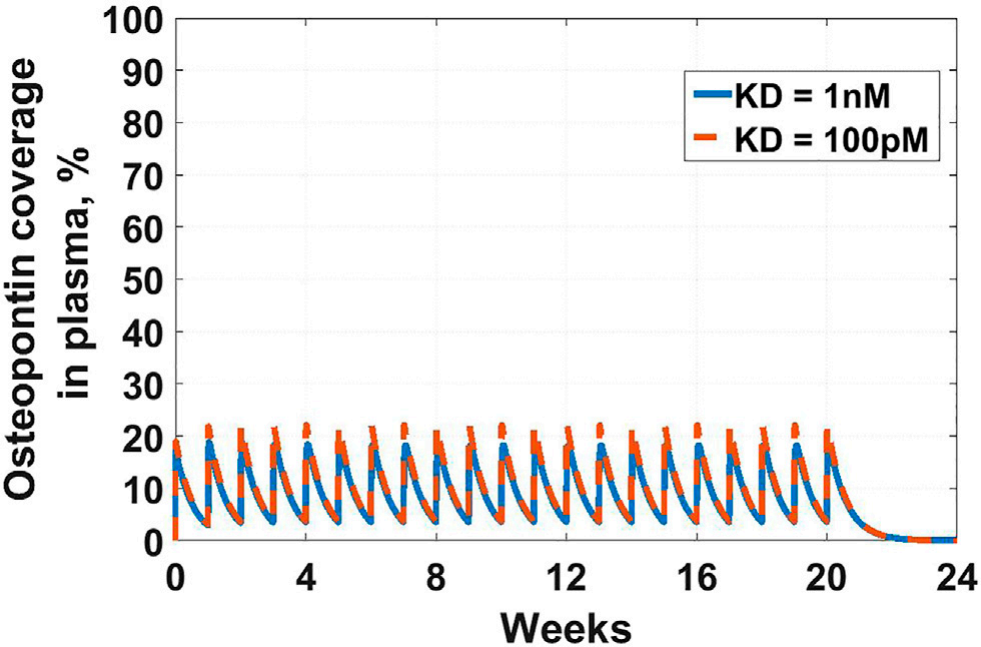
Osteopontin coverage in plasma: Shows the projected neutralization of osteopontin after mAb administration as described in *Osteopontin Example*. Simulated dose is 300 mg SC Q1W, with two antibody affinities – $K_{D}$ of 1 nM (solid blue line) and 100 pM (dashed orange line). Even peak projected neutralization is only 20%, which is unlikely to result in meaningful pharmacology.

**FIGURE 3:**
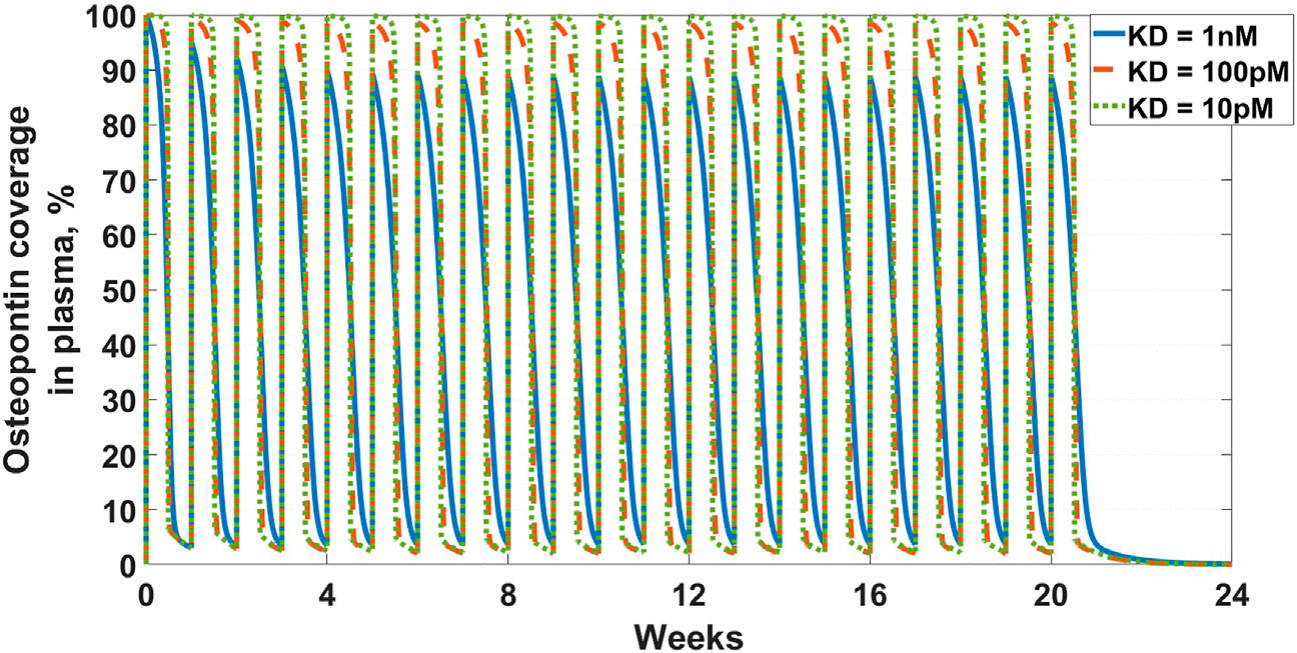
Osteopontin coverage in plasma: Shows the projected neutralization of osteopontin after mAb administration as described in *Osteopontin Example*. Simulated dose is 1,000 mg IV Q1W, with three antibody affinities – $K_{D}$ of 1 nM (solid blue line), 100 pM (dashed orange line), and 10 pM (dotted green line). All scenarios result in high peak neutralization, which is not sustained for the full duration of the dosing interval.

The first example in this section is an example of the latter-targeting osteopontin for rheumatoid arthritis (RA). Osteopontin is a secreted protein from a plethora of cells, that has been implicated in a variety of biological functions, from inflammation and fibrosis, to tumorigenesis and metastasis (Ashkar et al., 2000; Lund et al., 2013; Wang et al., 2014; Liu et al., 2015; Clemente et al., 2016). The goal of the work was to assess feasibility in suppressing osteopontin for the treatment of RA. A full SoA model was not utilized in this case because, as you will see, plasma levels of osteopontin were high enough to sufficiently inform feasibility, without the need for further modeling complexity. Target turnover was estimated using a human D3-leucine pulse-chase study similar to discussed in Estimating Target Turnover. Target serum concentrations were measured using a nano flow liquid chromatography-tandem mass spectrometry method similar to discussed in Estimating Target Concentrations. Mean serum concentrations were measured at around 10 nM and half-life was estimated at around 10 min. The scenarios presented here assume a mAb interacting with a soluble target and PK parameters for the drug are in Table 3. For the purpose of the example, to assess the degree of target coverage (free target reduction) in plasma, two dosing regimens were explored – 300 mg SC and 1,000 mg IV, both every week. These are not commercially viable doses for RA but were selected to explore the maximum attainable coverage with a monoclonal antibody targeting osteopontin. The effect of antibody affinity on target coverage was simulated using $K_{D}$ values of 1 nM, 100 pM, for both scenarios, and 10 pM for the IV dosing scenario. The results of the simulations can be seen in Figures 2 and 3. Ultimately, the high target levels and very fast target turnover resulted in low target trough coverage even at a non-commercially viable dosing regimens for RA. Drug affinity for the target was predicted to have little effect on the coverage, so affinity optimization would not help. Therefore, the target was determined undruggable with a regular monoclonal antibody and the project was not progressed. More detailed assessment of this target with a different modeling approach can be seen in Farrokhi et al. 2018b, where other antibody modalities were also explored.

**TABLE 3:** Antibody PK parameters for osteopontin feasibility analysis.

Parameter	Value (unit)	References
$V_{P}$	3.2 (L)	Betts et al. (2018)
$V_{2}$	2.2 (L)	
Cl	0.454 (L/d)	
Q	0.252 (L/d)	
$k_{a}$	0.26 (1/d)	Assumed, (Dirks and Meibohm, 2010)
F	60 (%)	

#### IL-33 Example

**FIGURE 4:**
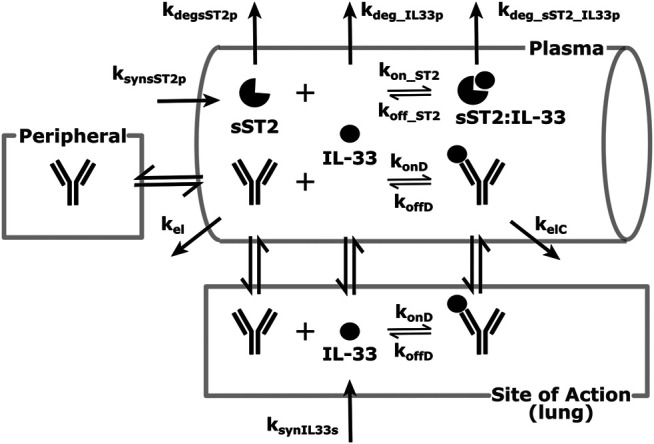
Modeling scheme of an anti-IL-33 mAb. The general processes are similar to the default scheme described in Figure 1, with several details adapted to the IL-33 scenario. IL-33 is synthesized at the SoA (lung) and distributes to the plasma. There, it can bind sST2 or get eliminated. sST2 is synthesized and eliminated in plasma only. The sST2:IL-33 complex clears in the plasma.

**FIGURE 5:**
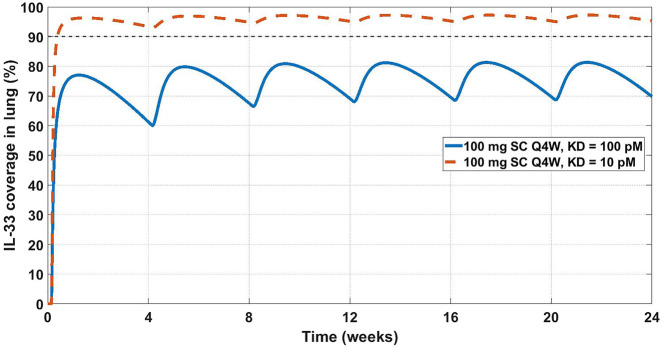
Projected IL-33 neutralization in the lung at 100 mg SC Q4W dosing at 10 pM (dashed orange line) and 100 pM (solid blue line) drug affinities. The 90% coverage line (dashed grey) is emphasized for convenience. The model projects that a 10 pM affinity would achieve 90% IL-33 neutralization in the lung.

Another feasibility example is IL-33. IL-33 is an alarmin, member of the IL-1 cytokine family, released by cells at the barrier surfaces (i.e., keratinocytes and airways epithelial cells) after disruption in the barrier function by pathogens, tissue injury, and cell death, and has been associated with atopic dermatitis and asthma (Saluja et al., 2015; Saluja et al., 2016). Asthma is the disease of choice for this example, therefore the SoA is lung. IL-33 signals through binding to ST2 and then forming a heterodimer with the IL-1 receptor (Saluja et al., 2015; Saluja et al., 2016; Griesenauer and Paczesny, 2017). ST2 can also be found in soluble form (sST2), which is a scavenger for IL-33 and constraints its signaling properties (Griesenauer and Paczesny, 2017). For the purpose of this example, our antibody competes with sST2 for binding to IL-33 in plasma. A scheme and description of the model is shown in Figure 4. The antibody binds IL-33 both in plasma and at the SoA. The distribution of the drug to the SoA and the periphery as well as assumptions regarding the mAb:IL-33 complex have been described earlier (see Drug Distribution Parameters). The target-related parameters and references used are described in Table 4. The mAb related parameters are described in Table 3 and Binding Parameters. $K_{D}$ for the purpose of this feasibility analysis was assumed to be either 100 or 10 pM - $k_{on}$ remained fixed (see *Binding Parameters*), while $k_{off}$ was calculated accordingly. Figure 5 shows projected target coverage at the site of action (lung). Based on the modeling results, the 100 mg SC Q4W dose is predicted to achieve greater than 90% neutralization of IL-33 at the site of action if the affinity of the mAb is closer to 10 pM than 100 pM. While a ∼10 pM affinity is challenging from an engineering perspective, design of a high-affinity antibody should be expected when targeting cytokines, especially if the ligand’s binding to its natural receptor is so tight (26 pM (Palmer et al., 2008)). In this case it was concluded, using the modelling analysis, that the target should be explored further, however extensive affinity optimization will likely be required to achieve sufficient neutralization. A Matlab Simbiology model file for this example is available in the Supplement section of this article.

**TABLE 4:** GSK3050002 two-compartment PK parameters (Bouma et al., 2017).

Parameter	Value (unit)	95% CI
$V_{P}$	3.63 (L)	3.44–3.83
$V_{2}$	3.19 (L)	2.89–3.52
Cl	0.475 (L/d)	0.439–0.514
Q	0.374 (L/d)	0.324–0.432

This early-stage feasibility analysis omits several potentially important aspects of the biology of IL-33, e.g., quick inactivation due to oxidation and proteolytic activities, and synthesis in cellular nucleus and release under inflammatory conditions (Cohen et al., 2015; Saluja et al., 2015; Griesenauer and Paczesny, 2017; Scott et al., 2018). The former can increase the apparent clearance of active IL-33 and both properties can skew the measurements of active free IL-33 in plasma and tissue. Also, considering the tight binding of IL-33 to sST2, further considerations can be made regarding the expression of membrane ST2 in the lung and the antibody’s interaction with the target in a full receptor:target interaction mechanistic modeling system. Potentially, a competing vs non-competing epitope may be important for enhancing target neutralization, which could be evaluated at the next stage of mechanistic modeling - right molecule. Several anti-IL-33 molecules have already been in the clinic, and a couple have shown positive results in asthma (Anaptysbio, 2014; Regeneron Pharmaceuticals, 2019), validating the model’s conclusions.

### Right Compound

Once feasibility has been established, the team delves deeper into assessing the molecular properties of the antibody necessary to neutralize the target. Most of the pharmacokinetic properties would depend on molecular assessment and there are currently few models that connect antibody molecular assessment and pharmacokinetics (Jones et al., 2019; Jones et al., 2020). Predominantly, modelers can assist the engineering team with projecting what antibody binding affinity is needed for the required level of neutralization (coverage). We use the next example of a clinical compound, to assess whether the mechanism was tested adequately and what affinity is required for improved target neutralization at a commercially viable dose.

**FIGURE 6:**
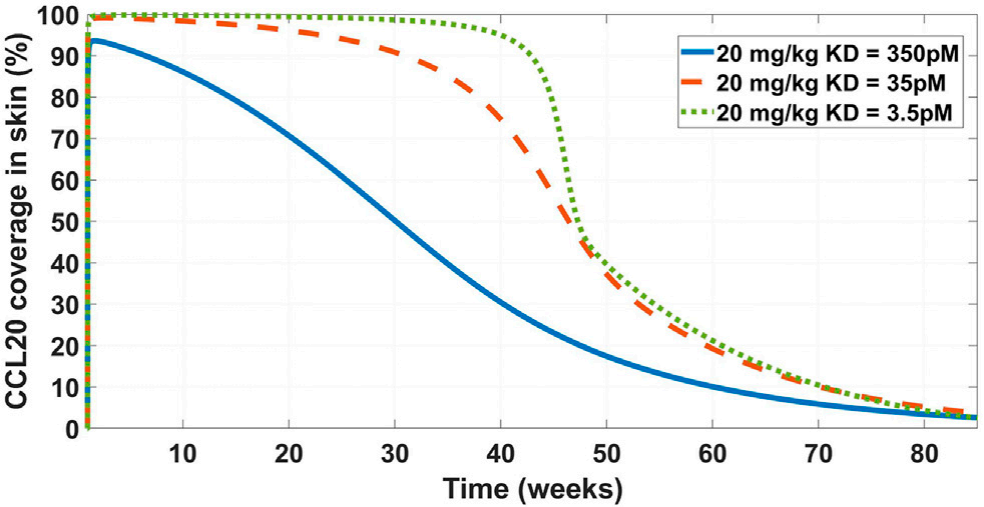
Model projected CCL20 coverage in the skin after administration of 20 mg/kg IV bolus dose of GSK3050002. Three mAb affinity scenarios were modeled – 350 pM (solid blue line), 35 pM (dashed orange line), and 3.5 pM (dotted green line). The low coverage at the base $K_{D}$ of 350 pM is consistent with observed lack of activity as described in Bouma et al. 2017.

**FIGURE 7:**
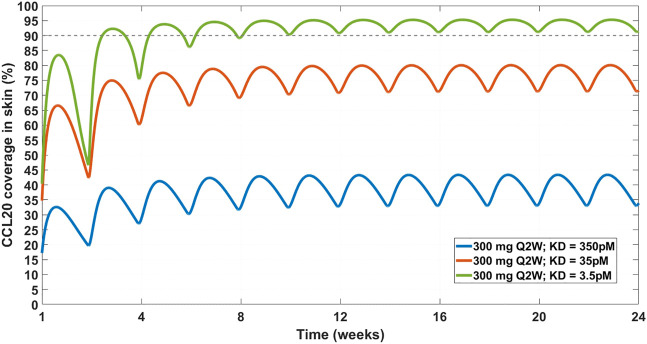
Projected CCL20 coverage in the skin at 300 mg SC Q2W dosing regimen and varying affinity. Three affinity scenarios were simulated – 350 pM (solid blue line), 35 pM (solid orange line), and 3.5 pM (solid green line). Thin dashed grey line indicates 90% coverage, which is emphasized for convenience. The model projects that a 3.5 pM affinity is required for achieving 90% CCL20 coverage in the skin.

Chemokine (C-C motif) ligand 20 (CCL20) is a chemoattractant for lymphocytes and dendritic cells in a variety of mucosal tissues (Schutyser et al., 2003). GSK3050002 is an anti-CCL20 monoclonal antibody that was tested in healthy volunteers (Bouma et al., 2017). The data presented in the study was drug, drug:target complex, and free target concentrations both in serum and in skin blister. The drug did not appear to inhibit monocytes and granulocytes activity in the skin blister model, so we decided to test whether a higher affinity antibody would be predicted to achieve higher and more sustained target coverage at the skin. For this purpose, a SoA model was constructed with skin as the SoA with the assumption that CCL20 was synthesized in the skin only and eliminated in the plasma only. Interstitial skin volume was assumed to be 1.125 L (Shah and Betts, 2012). Two-compartment PK model parameters (Table 4), $K_{D}$ (350 pM), target half-life (15 min), plasma:skin drug concentration ratio (20%, measured), and initial CCL20 concentrations in the plasma (30 pM) were fixed based data provided in Bouma et al., 2017. Skin concentrations of CCL20 are assumed to be 10-fold higher than plasma. Under these assumptions, even a dose of 20 mg/kg is not projected to achieve 90% target reduction in the skin for more than ∼ a week (Figure 6). At a regimen of 300 mg SC Q2W, the mAb is projected to need an affinity of 3.5 pM in order to reduce the target by 90% (Figure 7). This suggests that the affinity of the mAb was not tight enough and that the CCL20 mechanism was likely not adequately tested in the published clinical study.

While the general conclusion may still hold, several assumptions affect the results of the model. Some, like the synthesis of CCL20 in the skin, are reasonable given that the disease of interest is atopic dermatitis. The SoA:plasma ratio was assumed similar to the one found in IL-33 (see Table 5). That ratio would be target-dependent and potentially sensitivity analysis would need to be done to fully evaluate.

**TABLE 5:** List of target-related parameters used for anti-IL-33 model.

Parameter	Description	Value, units	Comments and references
IL33s_0	Initial concentration of free IL-33 at the SoA (lung)	11 pM	200 pg/mg of lung tissue in COPD or asthmatic patients (Cohen et al., 2015)
IL33p_0	Initial concentration of free IL-33 in plasma	1.5 pM	Assumed similar between asthma and allergic rhinitis – 27 pg/ml (Glück et al., 2019)
sST2_0	Initial concentration of free sST2 in plasma	27 pM	1 ng/ml in mild/moderate attack ((Oshikawa et al., 2001)), within two-fold of most other situations in Oshikawa et al. (2001) and levels in Glück et al. (2019)
sST2_IL33p_0	Initial concentration of sST2-bound IL-33 in plasma	$={\text{ k}}_{on\_ST2}\text{∗ IL}33{\text{p}}_{0}\text{∗}\frac{\text{sST}2{\text{p}}_{0}}{{\text{k}}_{off\_ST2}+{\text{ k}}_{\text{deg}\_\text{sST}2\_\text{IL}33\text{p}}}$	To preserve drug-free equilibrium values
IL-33 molecular weight	To convert mass concentration into molarity	18 kDa	Palmer et al. (2008)
sST2 molecular weight	To convert mass concentration into molarity	37 kDa	Mueller and Dieplinger, (2016)
k_deg_IL33p_	Degradation rate of IL-33 in plasma	4.2 1/day	∼4 h half-life in human lung explants (Cohen et al., 2015)
k_deg_sST2p_	Degradation rate of sST2 in plasma	2.6 1/day	6.3 h half-life (recombinant, IV administration) (Jacobs et al., 1993)
k_deg_sST2_IL33p_	Degradation rate of sST2:IL-33 complex in plasma	$={\text{ k}}_{\text{deg}\_\text{sST}2\text{p}}$	Assumed
k_on_ST2_	Association constant between IL-33 and sST2	358 1/nM/day	Palmer et al. (2008)
k_off_ST2_	Dissociation rate between IL-33 and sST2	$\;={\text{ k}}_{\text{on}\_\text{ST}2}{\text{∗ K}}_{\text{D}\_\text{IL}33\_\text{sST}2}$	K_D_IL33_sST2_ = 26 pM (Palmer et al., 2008)
k_ps_IL33_	IL-33 distribution rate from plasma to SoA	0.13 1/day	See Estimating Target Distribution
k_sp_IL33_	IL-33 distribution rate from SoA to plasma	$=\;\left({\text{k}}_{\text{ps}\_\text{IL}33\text{p}}\text{∗ IL}33{\text{p}}_{0}\right)\text{∗}\frac{{\text{V}}_{P}}{{\text{V}}_{\text{S}}\text{*IL}33{\text{s}}_{0}}+\frac{{\text{k}}_{\text{syn}\_\text{IL}33\text{s}}}{\text{IL}33{\text{s}}_{0}}$	To preserve drug-free equilibrium values
k_syn_IL33s_	IL-33 synthesis rate at the SoA	$=\;\left({\text{k}}_{\text{deg}\_\text{IL}33\text{p}}\text{∗ IL}33{\text{p}}_{0}+{\text{ k}}_{\text{deg}\_\text{sST}2\_\text{IL}33\text{p}}\text{∗ sST}2\_\text{IL}33{\text{p}}_{0}\right)\text{∗}\frac{{\text{V}}_{\text{P}}}{{\text{V}}_{\text{S}}}$	To preserve drug-free equilibrium values
k_syn_sST2p_	sST2 synthesis rate in plasma	$={\text{ k}}_{\text{deg}\_\text{sST}2\text{p}}\text{∗ sST}2{\text{p}}_{0}+{\text{k}}_{\text{deg}\_\text{sST}2\_\text{IL}33\text{p}}\text{∗ sST}2\_\text{IL}33{\text{p}}_{0}$	To preserve drug-free equilibrium values

### Right Dose

**FIGURE 8:**
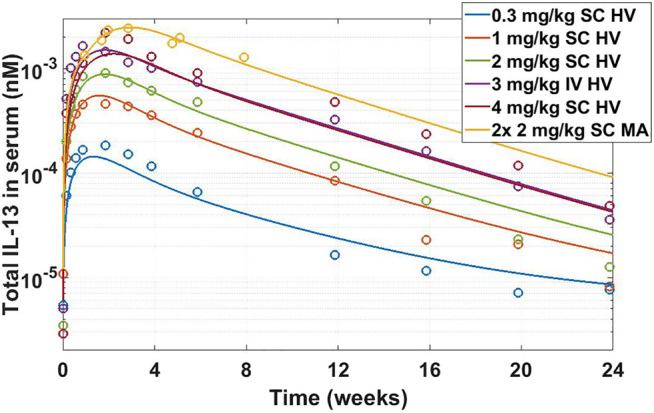
Total IL-13 accumulation after IMA-026 administration in Phase I trial - model simulations vs published data. Different color solid lines correspond to different doses, open circles with corresponding colors are clinical data. Labels: SC, subcutaneous; IV, intravenous; HV, healthy volunteers; MA, mild asthmatics. The model reasonably captures the behavior using internal affinity measures, previously published target data, and published PK parameters for IMA-026, without fitting any parameters.

Once drug properties have been established, modeling is utilized to project a clinical efficacious dose in different patient populations. This step is important both from standpoint of selecting doses for toxicology studies and assisting in dose selection for first-in-human studies. This particular example is a *retrospective* analysis of the clinical compound IMA-026, an antagonist monoclonal antibody against IL-13 (Gauvreau et al., 2011; Kasaian et al., 2011; Tiwari et al., 2016b). IL-13 is a cytokine with demonstrated role in many inflammatory diseases, including asthma. IMA-026 is an M1 type anti-IL-13 antibody (May and Fung, 2015), which blocks IL-13 from interaction with its receptors - IL13Rα1 (signaling receptor) and IL13Rα2 (decoy receptor) (Chandriani et al., 2014). IMA-026 data in healthy volunteers (NCT00517348) has been analyzed before (Tiwari et al., 2016b), where initial IL-13 concentrations, drug affinity, and target turnover were estimated. However, we demonstrate here that the accumulation of total plasma concentration of IL-13 can be obtained without fitting any parameters by using literature references (target turnover), pre-clinical observations (drug affinity), and relevant clinical data (PK and initial target concentrations). IL-13 target turnover was estimated to be around 20 min in mice (Khodoun et al., 2007), drug $K_{D}$ was 1 nM (based on internal measurements), median initial plasma IL-13 concentrations were estimated using ligand binding assay (LBA) to be around 0.06 pM for healthy volunteers and 0.12 pM for asthmatic patients, and PK parameters were estimated in Tiwari et al., 2016b. A SoA model was constructed with interstitial lung volume of 0.3 L (Shah and Betts, 2012) and SoA target concentrations of 0.03 pM in healthy volunteers and 0.4 pM in asthmatic patients (Kroegel et al., 1996). An average human bodyweight of 70 kg was assumed. Figure 8 shows the simulation of total IL-13 accumulation using the SoA model in plasma along with the observed clinical data from the healthy volunteer study. IMA-026 was evaluated further in an additional clinical study, NCT00725582, *Study Evaluating the Effect of IMA-026 on Allergen-Induced Late Asthma Response in Mild Asthma*. Two 2 mg/kg SC doses were administered 1 week apart.

**FIGURE 9:**
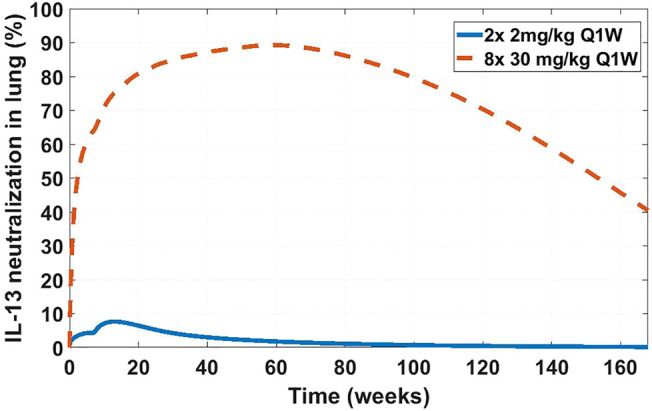
Projected IL-13 neutralization in lung at two dosing schedules for IMA-026. Two doses of 2 mg/kg a week apart (solid blue line) was the clinically tested dose in asthma patients (NCT00725582). Eight doses of 30 mg/kg a week apart (dashed orange line) is a hypothetical clinical dose at which the projected IMA-026 coverage reaches close to 90% IL-13 neutralization in the lung. Modeling suggests that the mechanism of IL-13 neutralization was likely untested in the clinic due to low tested dose, which is projected to result in low coverage.

The SoA model estimated that after 2 weeks dosing of 2 mg/kg SC Q1W the drug reached only around 8% target suppression at the site of action, while 8 weeks of dosing of 30 mg/kg SC Q1W would have achieved close to 90% coverage (Figure 9). Ultimately IMA-026 was abandoned since it did not show efficacy (Gauvreau et al., 2011). However, had data supporting an SoA model been available at time of the study conception, it could have projected the low coverage at 2 mg/kg and suggested either a longer duration study or a more appropriate dose for testing the mechanism. Likely, if SoA modeling had been available even earlier, an affinity maturation campaign could have been initiated to increase the affinity above 1 nM since a dose of 30 mg/kg Q1W is not commercially feasible. Suggestions for increased dose or improved affinity are corroborated by a crowd of anti-IL-13 monoclonal antibodies that have subsequently shown moderate to low efficacy in asthma (Gauvreau et al., 2011; Noonan et al., 2013; van Hartingsveldt et al., 2013; De Boever et al., 2014; Hanania et al., 2016), indicating the difficulty of achieving complete neutralization of IL-13 and/or its role as a standalone mechanism in the disease. The only anti-IL-13 mAb that has been in Phase 3 for asthma is lebrikizumab, with reported affinity of <10 pM (Ultsch et al., 2013). Lebrikizumab is currently in development for atopic dermatitis with positive results (Guttman-Yassky et al., 2020). Tralokinumab, whose affinity is reported at 58 pM (Popovic et al., 2017), has been approved for treatment of atopic dermatitis (LEO Pharma announces, 2021). The success and high affinity of both mAbs validate the model’s conclusions.

## Discussion

We have presented a three compartment mechanistic model that extends a typical two-compartment model by adding a site of action - a representation of the interstitial volume of the tissue where the interactions of the protein target with the drug are expected to contribute to disease modulation. The modeling is performed through a system of ordinary differential equations and is a mechanistic representation of the interaction of the drug with the target. The framework can be used for constructing a fit-for-purpose model to evaluate whether a target is biologically relevant and hitting the target is feasible (right target), guide drug properties for sufficient target engagement (right compound) and inform the necessary doses for achieving the degree of target engagement required for efficacy in the clinic (right dose). While the case studies described were categorized into examples of each of these three questions, these questions are inter-linked and in practice the modelling approach addresses all three if used at an appropriate stage of the drug discovery process. Ideally at project inception, a model should be generated to explore feasibility, affinity, and PK requirements for a desired dose level. Such an approach allows drug companies to focus on programs with the highest chance of success and limit the “wasted” resources on those which are more likely to fail. Hence, our proposal is to utilize this model in the early stages of drug discovery and, if possible, validate with clinical data.

The main feature of the SoA modeling approach is its flexibility. Depending on the target, a modeler can include any number of SoA compartments, ranging from zero to including every main tissue in the human body. Along with the system of equations, we suggest two key biomeasures: target concentrations and turnover, which represent essential parameters in early stage pre-clinical work. This is evident in the examples we presented - osteopontin could not be covered at any reasonable dose or affinity because of its high abundance and turnover; IL-33 needed high affinity for high neutralization but was deemed feasible due to relatively low expression and slow turnover; CCL20 also needed high affinity and a high dose to neutralize due to fast turnover. Different targets have different associated biology and will require different strategies to overcome difficulties in neutralizing them. Hence, while for the purpose of this discussion we are focused on the modeling structure, a capable biomarkers/biomeasures group is essential for the translational research effort.

Within the SoA model framework one can implement a variety of biological complexities: downstream or upstream biomarkers, different cell types, ligand-receptor binding, etc. The SoA model facilitates their implementations but one must weigh the complexity of the model versus the questions it tries to answer. The SoA modeling approach is not appropriate for a full disease model, for that goal a more complex QSP implementation would be appropriate. The SoA model is also not a physiologically based pharmacokinetics (PBPK) model - if the distribution of the drug in the whole body is important for the project, a PBPK model would likely be the appropriate tool.

The SoA model, as presented here, is fit for mAb modeling with its representation of mechanistic target binding and unbinding. The model can be adjusted and has been utilized to incorporate different molecular modalities - pegylated Fab fragments, bispecific antibodies, etc.

The examples in the manuscript are focused on soluble targets, which simplified some aspects of the presentation. Membrane-bound targets often present different challenges from modeling standpoint (i.e., target-mediated drug disposition, shedding of the membrane target) and relevant biomarker and biomeasures assays (i.e., number of receptors per cell, quantifying receptor internalization). These aspects can be described in a separate manuscript but there are excellent discussions on the topic, among which Aston et al. 2011 and Grimm 2009.

There are a number of shortcomings to the SoA modeling platform. The peripheral compartment may be underutilized - the model as presented here does not include target expression and turnover in the peripheral compartment or drug:target complex distribution in and out of the peripheral compartment. The method of fixing the ratio of drug concentration in plasma vs. the SoA assumes similar distribution to other previously measured antibodies. The rate of distribution of the drug into the SoA may have an effect on the target neutralization, and here it is calculated. Some of the mathematical methods may lead to non-physiological rates in order to preserve the measured concentrations at steady state. For example, the use of target distribution fixes the rate of distribution from plasma to the SoA and calculates the rate of distribution from the SoA into the plasma when assuming that the target is only synthesized in the SoA. This can lead to non-physiological differences in the plasma:SoA back and forth distribution rates. The very assumptions of target synthesis and distribution can alter the estimate of target suppression. However, despite these potential caveats of the base SoA model described in this manuscript, it is possible to adapt the framework to capture the relevant biological mechanisms as appropriate so that the sought physiological modulation can be described more accurately. Therefore, all the assumptions and calculations presented in this manuscript are just the most current iterations of ideas and are subject to scrutiny in the face of new facts and better representations.

As with all models, this framework requires validation. Early decisions can be made with sparse data and limited measurements but in order to improve confidence in the modeling results, ideally, measurements of key dynamics behaviors predicted by the model (longitudinal measurements of target engagement, free or total target levels, etc.) in relevant species with the candidate molecule or a suitable surrogate are needed for model validation. Furthermore, a retrospective validation using clinical data (external clinical data can also inform the pre-clinical model) should be performed when data is available in order to bridge the gap between theoretical and practical model projections. Some aspects of these validations include clinically-relevant disease-dependent level of target neutralization, distribution of the mAb into various types of SoA, or evaluation of the pre-clinical affinity biomeasures or functional assays and their translatability to the clinical setting. Not every project needs a site-of-action or a quantitative systems pharmacology model for successful translation from discovery to development. For the ones where understanding of the underlying pharmacology is limited, a simple exposure-response approach may be sufficient.

## Conclusion

Ultimately, the SoA platform model is a useful framework that has allowed us to inform the progression of many successful mAb programs. In particular, we have used the model to determine the doability of targets, drug requirements for “best in class” mAbs and dosing regimens to achieve required levels of target coverage to demonstrate efficacy. This modeling approach is fully integrated in the drug discovery process with the ability to make decisions believed to be high.

## Data Availability

Publicly available datasets were analyzed in this study. Available data is referenced in the article (literature references only).
